# Effect of Bright Light Therapy on Depressive Symptoms in Middle-Aged and Older Patients Receiving Long-Term Hemodialysis

**DOI:** 10.3390/ijerph17217763

**Published:** 2020-10-23

**Authors:** Shu-Yi Huang, Malcolm Koo, Tsung-Cheng Hsieh, Ru-Ping Lee, Huei-Chuan Sung

**Affiliations:** 1 Institute of Medical Sciences, Tzu Chi University, Hualien City, Hualien 970301, Taiwan; sc110@ems.tcust.edu.tw (S.-Y.H.); tchsieh@mail.tcu.edu.tw (T.-C.H.); fish@gms.tcu.edu.tw (R.-P.L.); 2 Department of Nursing, Graduate Institute of Long-Term Care, Tzu Chi University of Science and Technology, Hualien City, Hualien 973046, Taiwan; 3 Graduate Institute of Long-term Care, Tzu Chi University of Science and Technology, Hualien City, Hualien 973046, Taiwan; m.koo@utoronto.ca

**Keywords:** light therapy, older adults, depression, depressive symptom, hemodialysis, complementary medicine

## Abstract

Depressive symptoms are common psychiatric comorbidities among individuals receiving long-term hemodialysis. The aim of this two-arm parallel design study is to assess the effects of bright light therapy (BLT) on depressive symptoms among middle-aged and older adults receiving long-term hemodialysis. Study participants are recruited using convenient sampling from four dialysis clinics in eastern Taiwan. The eligible participants are block-randomized to either the BLT group (n = 30), with 30 min sessions of BLT five times a week for six weeks at their own home, or to the routine care control group (n = 30). The Beck Depression Inventory-II (BDI-II) scores and the salivary cortisol levels are obtained from the participants at three time points: baseline (T_0_), week 3 (T_1_), and week 6 (T_2_). The results, from the generalized estimating equations, indicate that the decline in the BDI-II scores over time is significant in the BLT group at T_1_ (β = −7.57, *p* < 0.001) and at T_2_ (β = −6.20, *p* = 0.002) compared to the control group. The decrease in salivary cortisol levels at each visit is also significant in the BLT group at T_1_ (β = −7.37, *p* = 0.017) and at T_2_ (β = −12.22, *p* = 0.005) compared to the control group. Our findings support the hypothesis that a six-week program of BLT is able to alleviate depressive symptoms in middle-aged and older patients who receive long-term hemodialysis.

## 1. Introduction

End-stage renal disease (ESRD), which refers to the permanent and irreversible damage to the kidneys, is the fifth stage in the progression of chronic kidney disease. Patients with ESRD must rely on renal replacement therapy, such as kidney transplantation or dialysis, for survival. It is estimated that over 2.6 million people receive renal replacement therapy worldwide and that the number is projected to double to 5.4 million by 2030 [1]. According to the 2017 United States Renal Data System’s annual report comparing data across 73 countries and regions, Taiwan showed both the highest prevalence of treated ESRD, at 3317 individuals per million general population, and the highest incidence of treated ESRD, at 476 individuals per million general population [2]. Although the direct health care costs of hemodialysis are covered by the Taiwan’s National Health Insurance system, which were estimated to be NTD 70,000 (USD 1 = NTD 30 in January 2020) per patient-month, there are additional out-of-pocket costs and productivity losses associated with hemodialysis, which have been estimated to be NTD 19,522 per patient-month [3].

Hemodialysis is a time-consuming process requiring two to three four-hour treatment sessions per week during a patient’s life time. Patients often experience a high level of physical, psychological, and social stresses, leading to an impaired quality of life. Common discomforts associated with hemodialysis include muscle weakness, tiredness, itchiness, thirst, joint pain, and sleep disruption [4,5,6]. In addition, depression is highly prevalent in hemodialysis patients [7], which is associated with dietary nonadherence [8], medication nonadherence [9], and even increased risk of suicide [10].

Although antidepressants are reasonably effective in treating depressive symptoms in patients with various chronic illnesses, relatively few studies have examined the safety and efficacy of treating depression in patients with ESRD. Impaired renal function can affect the pharmacokinetics of antidepressants, which in turn creates challenges for determining the optimal dose [11]. Therefore, various non-pharmacological approaches have been proposed as strategies to alleviate depressive symptoms in patients under hemodialysis treatment [12,13,14,15].

Bright light therapy (BLT) has been recognized as an effective, well-tolerated treatment for seasonal affective disorder [16]. Generally, only mild and short-lasting side effects, such as eyestrain and headache, have been reported in some studies, but not in others [17]. A randomized controlled pre-post intervention trial of 213 healthy young adults showed that a 30 min session of bright light exposure at 10,000 lux was not associated with a significantly higher incidence of any reported acute side effects compared to the placebo group receiving less than 500 lux red light [18]. Moreover, another study of 89 older adults who had major depressive disorders found no significant differences in any of the 28 possible adverse effects between the control group and the BLT group with three weeks of one-hour, early-morning, 7500 lux, bright light exposure [19]. In a study of 50 patients with seasonal affective disorder, no ocular abnormalities were observed after receiving either short-term or long-term (3–6 years) BLT treatment [20].

BLT has been explored for the management of depression associated with various conditions, such as non-seasonal depression [19,21], sleep disorders [22], bipolar disorder [23], pregnancy [24], and cancer [25]. BLT is thought to work through the activation of the hypothalamic suprachiasmatic nucleus (SCN) by nerve signals from specialized photosensitive ganglion cells in the retina. The SCN is the principal circadian pacemaker of the brain and is responsible for controlling the sleep-wake cycle, endogenous hormonal secretion, and core body temperature [21]. Because circadian rhythm disturbance is one of the hallmarks of mood disorders, correcting circadian disruptions by light cycle manipulations may therefore be able to alleviate depressive symptoms [26]. Until now, no studies have reported the effect of BLT on depression in individuals receiving long-term hemodialysis. Therefore, this study aimed to examine the effects of a six-week BLT intervention on depression in middle-aged and older adults receiving long-term hemodialysis.

## 2. Materials and Methods

### 2.1. Study Design and Participants

The protocol of the present study was approved by Medical Ethics and Institutional Review Board of Taoyuan General Hospital, Ministry of Health and Welfare, Taiwan (IRB No: TYGH105029).

A two-arm parallel design was used in this study. A fixed block randomization scheme with a block size of two was used to allocate participants to either an experimental group (BLT) or a control group receiving only routine care. Because BLT was performed in the participants’ own home, there was no mutual interference between the two groups.

Study participants were recruited using convenient sampling from four dialysis clinics in Hualien County, Taiwan, at two time periods (June–October 2017 and July–September 2018). The inclusion criteria included patients who: (1) had physician-diagnosed ESRD and were at the time receiving hemodialysis; (2) were 45 years old and older; (3) had received hemodialysis for a minimum of one month; (4) had a score of 14 or higher on the Beck Depression Inventory-Second Edition (BDI-II) at screening and baseline; (5) had provided written informed consent to participate. The exclusion criteria included patients who: (1) had diagnosed with visual lesions, such as cataract and glaucoma; (2) showed symptoms of acute pain or infection; (3) had difficulty in participating in the interviews.

### 2.2. Bright Light Therapy Intervention

BLT was administered using a Lightphoria 10,000 lux Energy Light Lamp (Sphere Gadget Technologies USA, San Francisco, CA, USA). The lamp was a portable light box 6 × 6.5 × 1 inches in size with an array of 72 eye-optimized light-emitting diode (LED) lights. According to the manufacturer, it delivers 10,000 lux of wide-spectrum non-flickering white light to simulate natural sunlight.

The participants in the BLT group were instructed to expose themselves to the light box five times a week for a total of six weeks, that each BLT session should last at least 30 min, and the sessions must be started within one to two hours of getting up in the morning. The main reason for scheduling the BLT session at such times is that previous research on seasonal affective disorder indicated that most patients become depressed due to a phase delay in circadian rhythms relative to the sleep/wake cycle [27]. Therefore, to elicit a phase advance, bright light exposure should be conducted in the morning. In addition, participants were asked to sit at a table with the light box placed at a distance of 30–45 cm in front of either side of their face.

### 2.3. Measurement Tools

The Beck Depression Inventory-Second Edition (BDI-II) is a widely used 21-item standard self-administered inventory designed to assess affective, behavioral, cognitive, motivational, and vegetative aspects of depression during the previous two weeks. Items are scored on a 4-point scale (0 to 3), ranging from 0 to 63, with higher scores indicating more severe depressive symptoms. A total score of 0–13 indicates minimal depression, 14–19 mild depression, 20–28 moderate depression, and 29–63 severe depression [28]. The Chinese version of the BDI-II has been shown to have good internal consistency (Cronbach’s α = 0.94) in a study of 180 Taiwanese psychiatric outpatients [29]. The BDI-II was administered to participants three times in this study, at the baseline prior to the intervention (T_0_), at week 3 (T_1_), and week 6 (T_2_).

Because dysregulation of the hypothalamic-pituitary-adrenal axis has been linked with depression [30], cortisol levels could serve as a marker to identify individuals at risk of depression [31,32]. Saliva samples from the participants were also collected at T_0_, T_1_, and T_2_. As cortisol displays a diurnal rhythm of expression, sample collection was performed within specific time windows of either 7:00–9:00 a.m. or 4:00–6:00 p.m. Once a participant chose a particular time window, the same window would be used in subsequent collections. The participants were told the day before the collection not to eat, drink (except water), or smoke within 30 min of sample collection. On the day of the saliva collection, the participants were asked if they had experienced any stressful events during the day of collection and, if affirmative, the saliva collection would be rescheduled. The saliva samples were collected using the passive drool method. Participants were asked to chew on a piece of sugar-free gum to increase salivary flow in the mouth and then to salivate through a straw into a vial. The collected saliva sample was temporarily stored in ice buckets filled with ice cubes and stored in a −20 °C refrigerator before being sent to the analytical laboratory (Redox Functional Medicine Laboratory, Taipei City, Taiwan). The free cortisol level in the saliva was quantified using an electrochemiluminescence immunoassay using the Elecsys cortisol reagent kit (Roche Diagnostics, Mannheim, Germany).

### 2.4. Statistical Analysis

Mean and standard deviation or frequency and percentage are used to describe the baseline characteristics of the participants, as appropriate. Baseline characteristics of the participants, between the BLT group and the control group were compared using the independent *t*-test for continuous variables and the Chi-square test for categorical variables.

To account for the covariance between observations on the same person at the three different time points measured in this study, generalized estimating equations (GEEs) were used [33]. The group × time interaction was used to assess the effect of BLT on the changes of BDI-II score and salivary cortisol level over time (T_0_, T_1_, and T_2_) compared to the controls. The unstructured working correlation matrix was applied in the GEE model to consider the time dependence of outcome variables in the working correlation matrix. All the analyses were performed by using IBM SPSS Statistics for Windows, Version 23.0 (IBM Corp, Armonk, NY, USA). All *p*-values were two-sided, with a value < 0.05 considered statistically significant.

## 3. Results

A total of 60 patients receiving long-term hemodialysis who met the inclusion criteria were recruited in this study. No side effects were reported by the participants during the study period. The mean overall age at the baseline was 62.0 years (range 45−78 years) and half of the participants were male. The BLT group (n = 30) and the control group (n =30) were not significantly different in their basic and clinical characteristics at the baseline, indicating that randomization was successful (Table 1).

**Figure 1:**
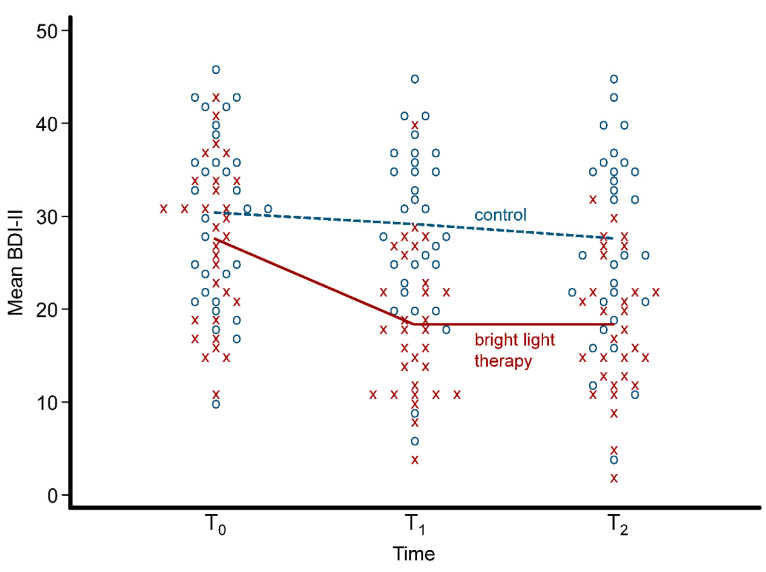
Dot plot with the mean of Beck Depression Inventory-Second Edition (BDI-II) scores in the bright light therapy group and the control group over the three time points. Each x symbol (red) represents an individual in the bright light therapy group and each o symbol (blue) represents an individual in the control group.

**Figure 2:**
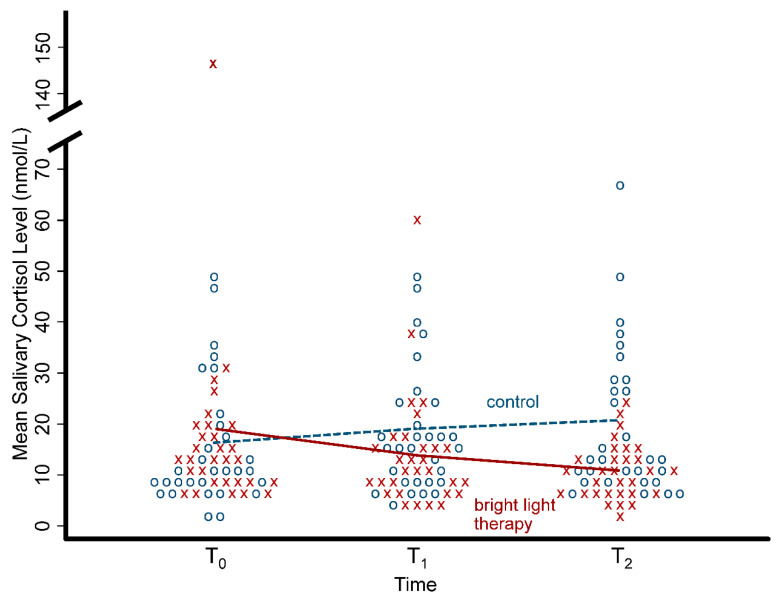
Dot plot with the mean of salivary cortisol levels in the bright light therapy group and the control group over the three time points. Each x symbol (red) represents an individual in the bright light therapy group and each o symbol (blue) represents an individual in the control group.

Table 2 shows the BDI-II scores and the salivary cortisol levels for the BLT group and the control group at each of the three time points of assessment. There were no significant differences in the mean scores of the BDI-II scores at the baseline (*p* = 0.199) between the two groups, but the mean BDI-II scores were significantly decline in the BLT group at both T_1_ (*p* < 0.001) and T_2_ (*p* < 0.001) compared with the control group. For the mean salivary cortisol level, there were no significant differences at the baseline (*p* = 0.558) and T_1_ (*p* = 0.158) but it was significantly reduced in the BLT group at T_2_ (*p* = 0.003) compared with the control group. All individual data for the BDI-II scores and the salivary cortisol levels are shown with a dot plot in Figure 1 and Figure 2, respectively.

Results from the GEE showed that the interaction terms between group and time were significant for both the BDI-II scores and the salivary cortisol levels at T1 and T2. In other words, the decrease in the BDI-II scores over time was significantly greater in the BLT group compared to the control group at T_1_ (β = −7.57, *p* < 0.001) and T_2_ (β = −6.20, *p* = 0.002); similarly, the decrease in the salivary cortisol levels was also significant in the BLT group at T_1_ (β = −7.37, *p* = 0.017) and T_2_ (β = −12.22, *p* = 0.005) compared to the control group (Table 3). As one individual in the BLT group had a very high salivary cortisol level (147 nmol/L) and could affect our results, we re-conducted our GEE with that particular individual removed. After the GEE had been re-conducted, the decrease in the salivary cortisol level was not significant in the BLT group at T_1_ (β = −4.53, *p* = 0.098) but remained significant at T_2_ (β = −7.70, *p* = 0.020) compared to the control group.

## 4. Discussion

In this study, we found that a six-week BLT program at home was able to significantly alleviate depressive symptoms in middle-aged and older adults receiving long-term hemodialysis. Both the BDI-II scores and the salivary cortisol levels in the BLT group showed significant improvement three weeks after the start of the intervention compared with the control group. The improvement remained significant six weeks after the start of the intervention. To the best of our knowledge, our study is the first to demonstrate the beneficial effect of BLT on depression in patients receiving long-term hemodialysis. Our results are consistent with other studies of BLT. A randomized placebo-controlled trial on 89 older patients with nonseasonal major depressive disorder showed that three weeks of one-hour early-morning BLT was able to significantly improve depression, as measured by the Hamilton Scale for Depression Score. Moreover, the evening salivary cortisol level significantly decreased in the BLT group compared with the placebo group. The effect of BLT was able to continue three weeks after discontinuation of treatment [19]. Another study on 27 pregnant women with nonseasonal major depressive disorder reported that 7000 lux fluorescent bright white light, administered in the morning, for one hour a day, for five weeks, could significantly improve depression more than the placebo 70 lux dim red light [24]. BLT was not found to be effective in alleviating depression in some conditions; for example, in a double-blind randomized controlled trial of 83 patients with Parkinson disease, a three-month BLT was not able to significantly reduce depressive symptoms compared with the control group [34]. A study on 34 older Taiwanese adults residing in a long-term care facility showed that exposure to a 10,000-lux light box for 30 min in the morning, three times a week, for four weeks was able to significantly reduce depression, as measured by the Geriatric Depression Scale-Short Form in the experimental group. However, no significant differences in the depression scores between the experimental group and control group were observed [35]. It appears that the effect of BLT on reducing depressive symptoms might vary between different conditions and the underlying mechanisms will require further investigation.

Cortisol hypersecretion has been suggested to play an important role in the pathophysiology of depression [36,37]. Our study showed that salivary cortisol level is significantly lower in patients receiving BLT. A randomized controlled trial in older adults with nonseasonal major depressive disorder also indicated that BLT could accelerate the diurnal decline in the salivary cortisol level [19]. Therefore, a possible mechanism of BLT on the reduction of depressive symptoms could be mediated through the hypothalamic–pituitary–adrenal axis and its regulation of diurnal cortisol secretion.

This study has a number of limitations. First, the participants were not blinded to group allocation. However, we included the salivary cortisol levels as an objective outcome in this study. Future studies may implement a placebo, such as a light box with a low level of luminance or a sham device with only a pilot light but no visible light, in the control group. Second, participants in the BLT group were instructed to start their session within one to two hours after getting up in the morning. Therefore, the effect of BLT administered at other times of the day could not be assessed and compared. Third, all participants were recruited from the eastern part of Taiwan and the study was carried out during summer months. Further studies in other parts of Taiwan and other seasons will be required to establish the generalizability of our findings. Fourth, we did not explore whether the effect of BLT was different between the sexes. Additional studies can be designed to address these issues.

## 5. Conclusions

Our findings revealed that a six-week program of BLT was able to alleviate depressive symptoms in middle-aged and older patients receiving long-term hemodialysis. Patients receiving long-term hemodialysis are often associated with depressive symptoms. Due to fatigue after hemodialysis, patients are less likely to engage in outdoor activities and are therefore unable to expose themselves to natural sunlight. The use of BLT as a simple and feasible approach to improve mood state through a readjustment of the circadian rhythm deserves further investigation.

## Figures and Tables

**Table 1:** Baseline and clinical characteristics of the participants in the bright light therapy group and the control group (*N* = 60).

Variable	Control (n = 30)	Bright Light Therapy (n = 30)	*p*
Age, year, mean (SD)	63.1 (9.1)	61.0 (9.4)	0.396
Sex			0.121
Male	12 (40)	18 (60)	
Female	18 (60)	12 (40)	
Educational level			0.108
Junior high school or below	22 (73)	16 (53)	
Senior high school or above	8 (27)	14 (47)	
Employed			0.754
No	24 (80)	23 (77)	
Yes	6 (20)	7 (23)	
Engaged in regular outdoor activity			0.796
No	15 (50)	14 (47)	
Yes	15 (50)	16 (53)	
Outdoor activity/week, number of times, mean (SD)	0.97 (1.27)	1 (1.14)	0.915
Time on hemodialysis, months, mean (SD)	95.0 (60.5)	87.5 (87.2)	0.700
Hypertension	21 (70)	15 (50)	0.114
Cardiovascular disease	6 (20)	7 (23)	0.754
Diabetes	17 (57)	15 (50)	0.605
Cerebrovascular disease	1 (3)	2 (7)	>0.999
Cancer	1 (3)	2 (7)	>0.999

SD: standard deviation. All values are *n* (%) unless otherwise stated.

**Table 2:** Beck Depression Inventory-Second Edition (BDI-II) scores and salivary cortisol levels for the bright light therapy group and the control group over time.

Variable	Mean (Standard Deviation)		*p*
	Control (n = 30)	Bright Light Therapy (n = 30)	
BDI-II Score			
T_0_	30.17 (9.33)	27.17 (8.53)	0.199
T_1_	28.90 (9.22)	18.33 (7.86)	<0.001
T_2_	27.37 (10.25)	18.17 (7.44)	<0.001
Salivary cortisol level (nmol/L)			
T_0_	16.05 (12.54)	19.07 (25.08)	0.558
T_1_	18.70 (12.19)	14.35 (11.39)	0.158
T_2_	20.14 (14.74)	10.93 (5.44)	0.003

BDI-II: Beck Depression Inventory-Second Edition (BDI-II); T_0_: baseline; T_1_: week 3; T_2_: week 6.

**Table 3:** Results of generalized estimating equations (GEEs) for the effects of bright light therapy on Beck Depression Inventory-Second Edition (BDI-II) scores and salivary cortisol levels.

Variable	Regression Coefficient (95% CI)	*p*
BDI-II Score		
Group (BLT vs. control)	−3.02 (−7.46, 1.46)	0.188
Time		
T_1_ vs. T_0_	−1.27 (−4.14, 1.61)	0.388
T_2_ vs. T_0_	−2.80 (−5.55, −0.05)	0.046
Interaction (group × time)		
BLT × T_1_ vs. control × T_0_	−7.57 (−11.63, −3.50)	<0.001
BLT × T_2_ vs. control × T_0_	−6.20 (−10.09, −2.31)	0.002
**Salivary cortisol level**		
Group (BLT vs. control)	3.02 (−4.47, 10.50)	0.430
Time		
T_1_ vs. T_0_	2.65 (−1.02, 6.32)	0.157
T_2_ vs. T_0_	4.08 (−2.56, 10.72)	0.259
Interaction (group × time)		
BLT × T_1_ vs. control × T_0_	−7.37 (−12.56, −2.18)	0.017
BLT × T_2_ vs. control × T_0_	−12.22 (−22.24, −2.20)	0.005

BDI-II: Beck Depression Inventory-Second Edition (BDI-II); BLT: bright light therapy; CI: confidence interval; T_0_: baseline; T_1_: 3 weeks; T_2_: 6 weeks.
